# The Role of the Primary Cell Wall in Plant Morphogenesis

**DOI:** 10.3390/ijms19092674

**Published:** 2018-09-09

**Authors:** Derek T. A. Lamport, Li Tan, Michael Held, Marcia J. Kieliszewski

**Affiliations:** 1 School of Life Sciences, University of Sussex, Falmer, Brighton BN1 9QG, UK; 2 Complex Carbohydrate Research Center, University of Georgia, Athens, GA 30602, USA; tan@ccrc.uga.edu; 3 Department of Chemistry and Biochemistry, Ohio University, Athens, OH 45701, USA; held@ohio.edu (M.H.); kielisze@ohio.edu (M.J.K.)

**Keywords:** morphogenesis, cell wall protein, hechtian oscillator, calcium signaling, H^+^-ATPase

## Abstract

Morphogenesis remains a riddle, wrapped in a mystery, inside an enigma. It remains a formidable problem viewed from many different perspectives of morphology, genetics, and computational modelling. We propose a biochemical reductionist approach that shows how both internal and external physical forces contribute to plant morphogenesis via mechanical stress–strain transduction from the primary cell wall tethered to the plasma membrane by a specific arabinogalactan protein (AGP). The resulting stress vector, with direction defined by Hechtian adhesion sites, has a magnitude of a few piconewtons amplified by a hypothetical Hechtian growth oscillator. This paradigm shift involves stress-activated plasma membrane Ca^2+^ channels and auxin-activated H^+^-ATPase. The proton pump dissociates periplasmic AGP-glycomodules that bind Ca^2+^. Thus, as the immediate source of cytosolic Ca^2+^, an AGP-Ca^2+^ capacitor directs the vectorial exocytosis of cell wall precursors and auxin efflux (PIN) proteins. In toto, these components comprise the Hechtian oscillator and also the gravisensor. Thus, interdependent auxin and Ca^2+^ morphogen gradients account for the predominance of AGPs. The size and location of a cell surface AGP-Ca^2+^ capacitor is essential to differentiation and explains AGP correlation with all stages of morphogenetic patterning from embryogenesis to root and shoot. Finally, the evolutionary origins of the Hechtian oscillator in the unicellular Chlorophycean algae reflect the ubiquitous role of chemiosmotic proton pumps that preceded DNA at the dawn of life.

## 1. Introduction

“If questions are to be asked about life processes, how can one fail to enquire into what is perhaps the most striking feature of life, morphogenesis? Morphogenesis is the end product of cell differentiation. Cell shape is one aspect of the more general problem of differentiation. Consider the primary cell wall, how it grows, and there is evidence enough for the primary wall playing a decisive role in influencing the shape of a cell. Therefore, the primary cell wall must be studied, but how?” (Lamport 1963) [1].

In 1952, Turing proposed that “morphogens acting together and diffusing through a tissue is adequate to account for morphogenesis.” His classic paper [2] described simple reaction–diffusion systems in mathematical detail, no doubt inspired by classic texts such as D’Arcy Thompson’s [3] “On Growth and Form” (1917), Wardlaw’s “Phylogeny and Morphogenesis” [4] and probably Joseph Needham [5]. However, despite the appealing simplicity of the theologian William of Occam (1287–1347) and his razor “entia non sunt multiplicanda praeter necessitatem”, the devil is in the biochemical details where “angels fear to tread”. Turing invoked Graham’s law of diffusion to infer the existence of morphogen gradients (that includes hormones) and the drift to equilibria analogous to an electronic oscillator, an idea that we recently developed as the Hechtian growth oscillator [6]. Despite Turing’s profound insight and subsequent progress, the problem of plant morphogenesis remains a huge challenge far exceeding the original formulation of simple diffusion. Here, we describe the origin of auxin, H^+^, and Ca^2+^ gradients and how their interaction creates morphogenetic patterns. We therefore approach the formidable problem of plant morphogenesis from an empirical biochemical perspective based on the dynamic role of AGPs (arabinogalactan glycoproteins) in a novel Hechtian growth oscillator. A cell surface AGP-Ca^2+^ capacitor is a crucial component that depends on the complex carbohydrate chemistry of classical AGPs [7,8] that chelate Ca^2+^ by paired glucuronic carboxyl groups of numerous small glycomodules. Not surprisingly, after their initial discovery as an arabinogalactan polysaccharide [9] and as a hydroxyproline-rich arabinogalactan polysaccharide protein complex [10], it has taken 60 years since the founder event [11] to unravel the structural basis for the proposed central role of AGPs in plant biology as a cell surface Ca^2+^ capacitor [12]. The role of AGPs emerged as an essential component of the Hechtian oscillator when we correlated tip-focussed cytosolic Ca^2+^ and tip-localised AGPs with Hechtian adhesion and rapid tip growth of pollen tubes [6].

Here, we extrapolate these recent results to morphogenesis and propose two new avenues: Firstly, we propose the avenue of cell walls and mechanoperception involving Hechtian transduction as described in detail here but not considered by Turing; secondly, we propose postulated chemical morphogen gradients [2] identified here as auxin and Ca^2+^. We propose that mechanotransduction generates both gradients, although each has a different biochemical origin; thus, auxin gradients originate from biosynthesis and transport by tissue-specific auxin efflux PIN proteins. On the other hand, cytosolic Ca^2+^ gradients originate from Ca^2+^ stored in a cell surface AGP-Ca^2+^ capacitor [12]. AGPs are essential components of the Hechtian growth oscillator that amplifies the magnitude and direction of stress vectors regulating plant growth [6]; that explains why plants have heavily invested in AGPs [13] and why this investment is so highly diversified [14]. Many AGPs are developmentally regulated and tissue-specific [15,16,17,18]. While the significance of a large pool of dynamic Ca^2+^ at the periplasmic cell surface seems inescapable to us, the role of AGPs in Ca^2+^ gradient formation in tissues is less obvious because it depends on a membrane-bound H^+^-ATPase that dissociates AGP-Ca^2+^. That shows the close link between auxin and Ca^2+^ signalling with the primal proton pump. Paired glucuronic carboxyls of AGP glycomodules bind Ca^2+^ tightly [12] (analogous to Ca^2+^ chelation by dicarboxylic acids). This effectively scavenges less tightly bound Ca^2+^ of pectin and also free Ca^2+^ in the transpiration stream. Thus, abundant cell surface AGP-Ca^2+^ acting as a Ca^2+^ sink would enable both cells and tissues to compete for Ca^2+^, so that Ca^2+^ supply meets demand where it is greatest, such as in meristem primordia. The “choice” of Ca^2+^ as a universal signalling ion can be ascribed to the size (0.9 Å) of its ionic radius; thus, Ca^2+^ sheds water more rapidly and is therefore more reactive than other divalent ions—notably Mg^2+^ [19]—of similar charge but with a smaller ionic radius (0.65 Å) that binds the water of hydration more strongly than Ca^2+^.

The following five sections of this essay show how morphogenetic patterns originate, involving (1) cell wall mechanotransduction and the developmental sequence, (2) the embryogenesis of the fertilised egg cell, (3) roots, (4) shoots, and finally (5) the evolution of morphogenesis.

Most models of morphogenesis, particularly computer-generated models, generally favour a genetic perspective with an emphasis on signalling cascades. However, they ignore two “unknown unknown” missing pieces of the morphogenetic puzzle, identified here as Hechtian mechanotransduction and the AGP-Ca^2+^ capacitor.

## 2. Cell Wall Mechanotransduction

**Figure 1:**
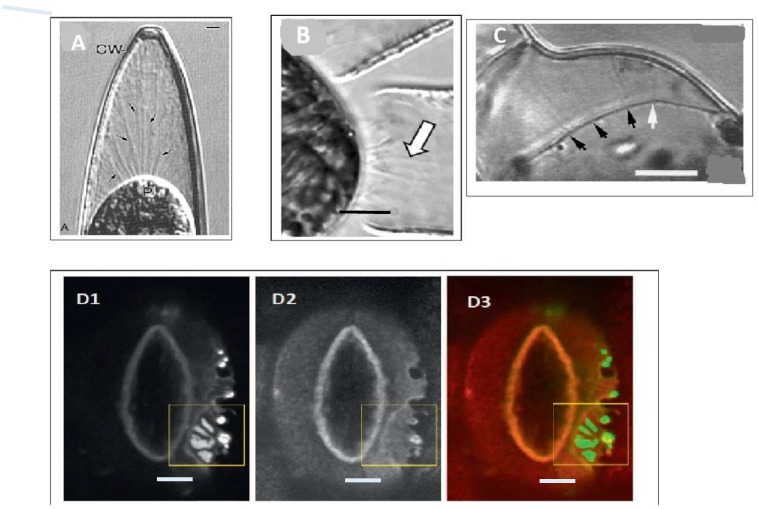
Hechtian strand occurrence: from algae to chloroplast guard cells. (**A**) Closterium desmid in 12% sucrose: reprinted from [22], Scale bar = 2.1 µm; (**B**) *Penium margaritaceum*: reprinted from [23], Scale bar = 2 µm; (**C**). Ginkgo cells plasmolysed in 0.3 M NaCl reprinted from [24], Scale bar is 10 µm; Arrows in (**A**–**C**) show location of Hechtian strands. (**D1–3**) Guard cell H^+^-ATPase and its translocator PATROL (proton ATPase translocation control) in *Arabidopsis*. The plasma membrane ATPase AHA1 and its translocator PATROL1 co-localise at the tips of Hechtian strands in plasmolyzed guard cells transformed with GFP-PATROL1 (**D1**) and RFP–AHA1 (**D2**) merged in (**D3**). Enlarged images of the region enclosed in the yellow square are not shown here. Reprinted from [25], Scale bars = 5 µm.

Hecht’s classical observations of adhesion between wall and plasma membrane in plasmolysed cells [20] and its significance to cell signalling have been overlooked for more than a century with few exceptions [21]. This seems inexplicable and is most likely because the emphasis has been exclusively on signalling molecules rather than a physical connection. Widespread Hechtian adhesion, which is prominent particularly during rapid tip growth, after plasmolysis (Figure 1) suggested the presence of specific tethers that transduce the stress–strain status of the wall to plasma membrane receptors that can respond to piconewton (10^−12^ N) forces (Table 1). 

However, the Hechtian transduction hypothesis creates three dilemmas:

1. How does it distinguish between the huge forces (mega Pascal range) exerted on the membrane by osmotic pressure and the minute piconewton forces that activate Ca^2+^ channels and ATPases?
2. How does it provide a directional signal in response to anisotropic stress? [26]
3. Why does the wall need strong adhesion to the plasma membrane although turgor pressure ensures it? Is the elusive mechanosensor a Boojum or a Snark?

Hechtian adhesion must satisfy several criteria: minimally, it must involve strong, stable and specific interactions with both the cell wall and plasma membrane. Several candidates frequently suggested such as wall-associated kinases and integrins lack hard data and can be eliminated. For example, integrins recognise the RGD motif of the mammalian extracellular matrix but are absent from plants. Other possible candidates include formins and many AGPs anchored securely to the plasma membrane [27,28,29,30] by a C-terminal GPI-lipid double tail of long-chain fatty acids that requires a force of ~350 piconewtons to detach from the membrane [31], compared with changes in molecular conformation that are sensitive to far fewer piconewtons [32,33].

At-AGP57C (At3g45230) is covalently linked to pectic RG-I homogalacturonan [34] and is also likely anchored to the plasma membrane by its predicted GPI signal sequence. This identifies a putative Hechtian adhesion site of crucial significance that would enable the instant transmission of the wall stress–strain status and its rate-determining pectin rheology to enable the rapid tip growth of root hairs and pollen tubes. 

Wall stress and strain is thus focussed at the molecular level on Ca^2+^ channels and H^+^-ATPase; both membrane components are stretch-activated by just a few piconewtons. 

**Figure 2:**
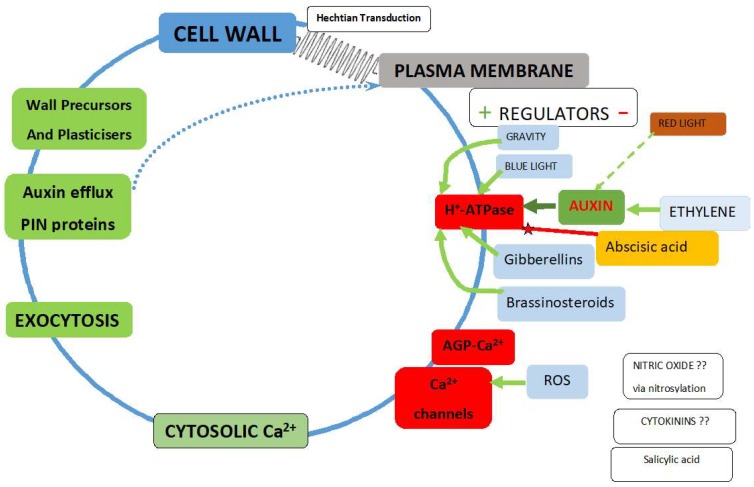
The Hechtian growth oscillator. Regulators of the plasma membrane H^+^-ATPase reflect its two major roles: 1. It maintains negative inner membrane potential and enhances anion exit and cation entry; 2. It dissociates AGP-Ca^2+^ as a source of cytosolic Ca^2+^. Green arrows indicate upregulation or red represent downregulation, with exceptions where the mechanism remains to be elucidated. Auxin activates H^+^-ATPase. cf. Figure 3: Plasma membrane H^+^-ATPase regulation is central to plant biology [38]; the effects of high steady state auxin levels on root cell elongation in Brachypodium [39]; auxin-driven morphogenetic patterns depend on unidirectional fluxes [40]; the evolution of auxin signaling and PIN proteins [41]; what initiates auxin biosynthesis remains unknown [42]; auxin activates the plasma membrane H^+^-ATPase via phosphorylation [38]. Abscisic acid negative regulation: decreases steady-state levels of phosphorylated H^+^-ATPase possibly by promoting dephosphorylation via a protein phosphatase [43] and suppresses hypocotyl elongation in Arabidopsis [43]; abscisic acid stress signalling evolved in algal progenitors [44]. Blue light: the blue light photoreceptor pigment phototropin increases cytosolic Ca^2+^ [45]. Brassinosteroids: Increase cytosolic Ca^2+^ via increased auxin levels [46]. Cytokinins enhance cell division by unknown mechanisms. Ethylene upregulates auxin biosynthesis in the Arabidopsis root apex and inhibits root cell expansion [47]; thus, anthranilate synthase mutants yield ethylene-insensitive root growth phenotypes. Ethylene specifically inhibits the most rapid growth phase of expanding cells—normally the root hair initiation zone but ethylene moves it much closer to the tip. Auxin and ethylene act synergistically to control root elongation, root hair formation, lateral root formation and hypocotyl elongation [48]. Ethylene modulates root elongation through altering auxin transport: Ethylene binds to receptor proteins such as ETR1 and EIN2 controlling transcription factor EIN3 that targets ERF1, the ethylene response factor that regulates diverse genes, most likely tissue-specific. In shoots, auxin moves from the apex to the base [49]. PIN1 mutants decrease auxin transport in inflorescences while PIN2, PIN3, and PIN7 mutants decrease polar auxin transport in roots. In roots, auxin moves rootward via PIN1, PIN3, and PIN7 in the central cylinder and shootward in the outer cell layers via PIN2 which mediates gravitropism. Ethylene-induced auxin synthesis involves the α and β subunits of anthranilate synthase [47]. Fusicoccin: The fungal toxin fusicoccin fills a cavity in the interaction surface between PM H^+^-ATPase and 14-3-3 proteins to form a tight bridge that activates the ATPase irreversibly [38]. Gibberellins increase cytosolic Ca^2+^ via a DELLA-independent signalling pathway [50]. Gravity: in root gravitropism, auxin regulates root curvature via apoplastic pH and a Ca^2+^-dependent signalling pathway [51]; PIN proteins responsible for polar auxin transport and gravitropism reviewed in [42]; in microgravity, cucumber seedling PIN protein distribution is parallel to the minor root axis. However, a 1 g force re-aligns PIN1 to the lower side of the endodermis thus facilitating auxin transport from the upper side of the root to the lower side. Similarly, PIN3 and PIN7 of the gravity sensing columella also re-align to the lower side [52]. Nitric oxide: high apical levels of reactive oxygen species (ROS) in *Arabidopsis* root hairs suggest possible activation of a Ca^2+^ channel that modulates root hair-tip growth [53]. Red light: the elevation of auxin levels is well established as an early event, in response to response to prolonged shade. During an initial triggering phase, phytochrome interacting factors (PIFs) bind to the promoters of auxin synthesis genes and generate a burst of auxin that promotes growth [54]. ROS: NADPH oxidase generates active oxygen species that activate Ca^2+^ channels and regulate cell expansion and root morphogenesis [55].

Piezo proteins responsive to mechanical force are well-documented in animal systems [33]. Finally, Hechtian stress–strain transduction and exocytosis comprise the novel Hechtian oscillator [6], an exquisitely designed high gain biological amplifier (Figure 2) that translates piconewton forces [37] into tropisms, with wide ramifications in plant biology.

Although osmotic pressure is equally distributed at the plasma membrane, wall pressure exerts an equal and opposite force. However, a direct covalent connection between pectin and the plasma membrane would enable instant transmission of the wall stress–strain status by fibrous macromolecules.

Two extreme examples, stomata and pollen tubes, demonstrate the efficacy of Hechtian adhesion: the stomatal guard cell wall elasticity transmits stress while the rapid tip growth of a pollen tube involves wall plasticity; when stretched, it transmits strain arguably via AGP57C [34] or its homologous GPI-anchored classical AGPs, supporting a vital role for the Hechtian growth oscillator in morphogenesis as discussed in subsequent sections.

The pollen tube tip has the simplest primary cell wall—almost exclusively pectin and with fast tip growth—so is an ideal system to test hypotheses involving the Hechtian transduction of wall stress–strain. Pectin rheology is deceptively simple and depends largely on the degree of methyl esterification of pectic polygalacturonic acid residues, in contrast to AGP glucuronic acid, which is never esterified. Highly methyl-esterified pectins form gels, while de-esterification followed by Ca^2+^ crosslinking forms much stronger gels. A frequently cited pectic paradigm [56] assumes sufficient available free Ca^2+^ for crosslinking but ignores the regulation of Ca^2+^ in muro by AGPs which, with their demonstrated higher affinity for Ca^2+^, compete for pectic Ca^2+^. Such competition effectively strips Ca^2+^ from pectin. The concomitant electrostatic repulsion of negatively charged pectic carboxylates explains the increase in wall plasticity by pectin methylesterase [57]. This effect, not previously considered, may also explain the role of the small diffusible “AGP peptides” [58] that possess two arabinogalactan glycomodules, presumably with paired glucuronic acid residues that bind Ca^2+^. Significantly, small AGP peptides are upregulated by auxin [39], suggesting another subtle level of control over plasticity of the pectin-rich primary cell walls. 

Driven by turgor pressure, cells expand in a direction orthogonal (i.e., at right angles) to the direction of stress that strains the pectic matrix; the concomitant Hechtian transduction of pectic strain initiates Ca^2+^ oscillations and exocytosis (Figure 2). The stress vector also causes the orthogonal orientation of microtubules and cellulose deposition [26] that reinforce the pectic matrix. As noted above, pectin rheology depends on its methyl esterification status regulated by pectin methyl esterase [59], the availability of free Ca^2+^ and borate crosslinking of the RG-II pectic component. Crosslinking of the cellulosic component (notably absent from pollen tube tips) may also be regulated by expansin [60,61].

Such complexity explains why the molecular basis of cell wall plasticity remains recalcitrant since first described [62]. “The difficulty lies not in the new ideas, but in escaping from the old ones” (John Maynard Keynes). Many are Boojums—hypotheses that postulate non-existent entities are not falsifiable and should disappear: for example, the widely accepted effect of low pH on wall loosening by activating enzymic cleavage of load-bearing covalent crosslinks. However, both crosslinks and enzymes remain unidentified. In the absence of an alternative viable unifying hypothesis, the Hechtian oscillator emphasises the role of Ca^2+^ in the exocytosis of wall plasticisers as detailed in [6] rather than covalent bond cleavage. Plant cells share a wall with neighbours and can therefore use Hechtian adhesion to sense which wall is being highly stressed by a rapidly expanding neighbour cell. Such stressed cells respond by the rapid reorientation of auxin efflux PIN proteins to direct auxin efflux towards their rapidly expanding neighbour [26]. Cells also compete for available Ca^2+^ by increasing the size of their cell surface AGP-Ca^2+^ capacitor; this can increase the amplitude of Ca^2+^ oscillations, thereby enhancing exocytosis.

Consider the primary receptors of force transduction. What are they, and how do they propagate tension to the cell interior? Stretch-activated Ca^2+^ channels [63] and auxin-activated H^+^-ATPase [64] control Ca^2+^ influx as follows: the plasma membrane ATPase [38,65] is similar to the mitochondrial ATP synthase: a rotary nanomachine fuelled by the proton motive force that generates ATP. However, in reverse, fuelled by cytosolic ATP, it becomes a molecular turbine rotating at up to ~8000 rpm, ejecting proton jets that pump about three protons into the periplasm for each ATP molecule hydrolysed [66].

These powerful proton jets dissociate the AGP-Ca^2+^ of the cell surface capacitor that supplies the Ca^2+^ channels. The evidence [6] for this is a 100% correlation between tip-localised AGPs and “tip-focussed” cytosolic Ca^2+^ during the rapid tip growth of pollen tubes.

How Ca^2+^ fluxes translate into morphogenesis depends on the local wall plasticity that determines the direction and magnitude of the wall stress vector; its amplification depends on the size of the AGP-Ca^2+^ capacitor (Figure 2) and its obvious abundance at metabolically active sites [67]. Arguably, the stress vector also determines the anisotropic location of Hechtian adhesion sites that activate the local vectorial exocytosis of wall precursors and dynamic reorientation of auxin efflux (PIN) proteins. Mechanical stress causes auxin efflux carrier localisation at the plasma membrane adjacent to the most stressed or strained walls cells and thus directs auxin toward neighbours that are rapidly expanding [68]. Counterintuitively, “the net flow of auxin in shoot tips is up the auxin gradient such that any cell directs its auxin toward neighbouring cells that have a higher auxin concentration” [26]. Auxin flux against the concentration gradient towards regions of active expansion growth, although an apparent contravention of the Second Law of Thermodynamics, is pithily summarised by Mathew’s Law: “To him that hath shall be given. And to him that hath not shall be taken away”.

Plant cells exploit a “thermodynamic loophole” based on their ability to trap ionised auxin in the cytosol (pH 7.4) where it cannot freely permeate the plasma membrane. However, in the wall at ~pH 5, auxin is protonated and thus freely permeates the plasma membrane of adjacent cells. Thus, auxin wins the Sysiphean uphill struggle.

The regulation of the oscillator at several biochemical levels involves Ca^2+^ channel activity and the plasma membrane H^+^-ATPase (Figure 2). The proximity of a large or small AGP-Ca^2+^ capacitor and its dissociation by ATPase proton pump thus creates dual gradients of auxin and Ca^2+^; coincidentally, two interacting morphogens required for the creation of “Turing patterns” [2]. By connecting these gradients, both dependent on the proton motive force, the Hechtian oscillator emerges as a master regulator of plant growth; this paradigm shift demands the reappraisal of the acid growth hypothesis [69] that depends on the widely accepted activation of wall-loosening enzymes. However, alternative explanations for a low wall pH include the PIN-directed auxin transport of protonated auxin and increased plasticity of deprotonated pectin. Finally, the release of Ca^2+^ from AGP-Ca^2+^ by a proton pump [12] is an extrapolation of Mitchell’s original chemiosmotic hypothesis [70].

## 3. Embryogenesis

**Figure 4:**
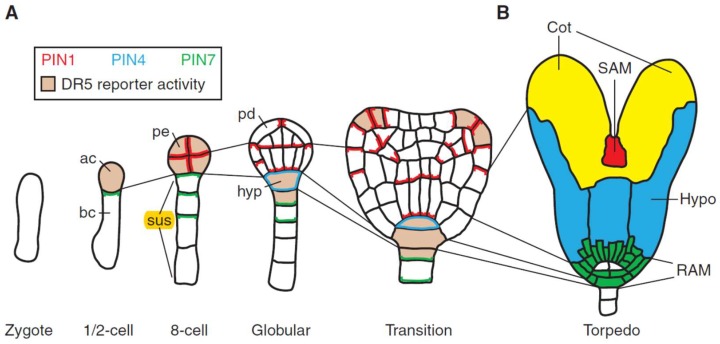
Embryogenesis in Arabidopsis: Cell lineages, PIN protein localization, and auxin response maxima (reprinted from [71]). Auxin response maxima and PIN protein localization follow a regular cell division pattern. Thin lines show lineages between stages: PIN protein localization is shown as follows: red (PIN1), blue (PIN4), and green (PIN7), and DR5 reporter is pink. (**A**) After the division of the zygote, one and two-cell embryos express PIN7 in the basal daughter cell (bc), and the apical cell (ac) expresses the DR5 reporter. After two more cell-divisions, all proembryo (pe) cells express PIN1 and DR5 reporter. Basal suspensor (sus) cells express PIN7 localized on the proembryo side. At the globular stage, central lower cells of the proembryo establish basal PIN1 polarity while PIN1 localizes apically in outer protoderm (pd) cells. At the same time, PIN7 polarity reverses in suspensor cells and PIN4 is activated in the uppermost suspensor cell that now expresses the DR5 reporter and is specified as hypophysis (hyp). During the transition stage, the PIN1 polarity at the flanks of the apical embryo half converges in adjacent cells, accompanied by the appearance of new DR5 maxima. These sites mark the initiation of the cotyledons; (**B**) The torpedo stage shows discrete regions of the embryo: RAM (root apical meristem), green, root apical meristem (white, future quiescent centre); hypo, blue, hypocotyl; Cot, yellow, cotyledons; and SAM (shoot apical meristem), red, the shoot apical meristem.

The above description of stress-dependent auxin and Ca^2+^ gradients shows how polarity can arise at the earliest stages of embryogenesis, particularly with an AGP-Ca^2+^ sink as a source of dynamic cytosolic Ca^2+^, while tissue tensions determine the orientation of auxin efflux proteins and cellulose deposition. Organogenesis then follows similar biochemical and biomechanical rules. Every budding botanist sees sliced celery stalks curl because internal tissues are under compression and outer tissues under tension. This simple principle applies during embryogenesis as the stress vector changes from the internal meristem (compression) to the outer protoderm (tension). The concomitant rearrangement of auxin efflux proteins in the protoderm under tension results in lateral auxin export from its apex outwards, with consequential slow growth and anisotropy at the torpedo stage that splits the tissue into the two cotyledons [71] (Figure 4). 

Angiosperm double fertilisation results in a triploid endosperm and a diploid zygote that elongates and establishes the shoot–root hierarchy with an initial asymmetric division into a small apical cell that becomes the proembryo; the large vacuolated basal cell divides by transverse divisions to produce the suspensor, a short file of cells that act as an “umbilicus” connecting the embryo with maternal tissues. The hypophysis is a remarkable pivotal apical cell of the suspensor, and as the precursor to the embryonic root, it demarcates the crucial zone between shoot and root. Thus, many auxin mutants result in a defective hypophysis and rootless seedlings. Presumably, a simplified morphogenetic programme of hypophysis silences most major shoot programmes and defines the fate of future cells in the root as a “partial shoot” [72] characterised by the root tip meristem, xylem, phloem, pericycle and lateral roots.

Plant cells are generally totipotent, and single cultured cells can regenerate an entire plant [73,74] While cell lineage does not necessarily determine cell fate, the aphorism of Hans Driesch that “the fate of a cell is a function of its position” implies that both its lineage [75] and position [76] are pertinent. Indeed, “morphogen” gradients evident as auxin and AGP-Ca^2+^ are highly correlated with the earliest stages of embryogenesis including the polarised haploid egg cell that yields a zygote with polarised AGP distribution increasing at the later two-cell stage and onwards [77]. Auxin biosynthesis evidenced by the DR5 reporter is similarly localized, particularly at the 2-cell, 8-cell and globular stages in *Arabidopsis* [71]. Auxin distribution depends on auxin efflux carriers which are initially inferred [78] and subsequently identified at the genomic level by “PIN” mutants that have phenocopied inhibitors of auxin transport [79]. Unlike the anisotropic protoderm, the early growth of meristematic tissue is isotropic. Variations in the local growth of the meristem arise possibly because cell surface AGPs compete for limited Ca^2+^; combined with tissue tensions, this leads to boundary regions which are low in Ca^2+^. These peripheral anisotropic regions, particularly in the ectoderm, result in tge rearrangement of auxin efflux proteins with consequent auxin depletion in boundary zones between the meristem, hence the origin of new primordia and phyllotaxis; i.e. auxin peaks generate a spiral phyllotactic pattern. 

Much work over more than 25 years (Table 2) reveals a remarkable family of tissue-specific auxin efflux (PIN) proteins that ensure a regulated supply of auxin during embryogenesis and the ensuing stages of morphogenesis. However, until now, the signals that initiate PIN protein localisation remain unknown; the Hechtian transduction proposed here is the best candidate based on considerable supporting evidence.

The Hechtian growth oscillator generates morphogenetic patterns; arguably, these depend on localised adhesion sites that result in vectorial exocytosis of wall precursors. This includes auxin efflux PIN proteins directed to the sites of the highest strain in expanding cells, [26] thus ensuring a supply of auxin for their continued growth. AGPs specifically localised in the suspensor [81] (Figure 4 in reference [81]) (6 to 9 cells in *Arabidopsis*) with apically polarised PIN4 and PIN7 auxin efflux proteins [80] highlight its active role as an “umbilicus” supplying auxin and nutrients from maternal tissues to the embryo.

Stress also predicts the plane of cell division marked by the preprophase band of microtubules [82] as putative mitosis stabilisers [83] and the appearance of the cell plate templated by extensin self-assembling amphiphiles; [84] hydroxyproline-rich glycoproteins central to plant growth once again cooperate with biomechanical forces. Elegant laser ablation experiments alter the orientation of PIN proteins in the apical meristem [26]. These classic experiments [16,26,85] show that tissue biomechanics create stress vectors that reorientate tissue-specific auxin efflux PIN proteins. We infer that specific Hechtian adhesion is the “missing link” that connects biomechanical stress vectors with auxin action and PIN reorientation: a chemically defined covalent connection between cell wall pectin and a known plasma membrane such as AGP57C likely transduces stress that dissociates AGP-Ca^2+^, thus increasing cytosolic Ca^2+^ that triggers vectored exocytosis. We infer that Hechtian adhesion and AGPs amplify both the direction and magnitude of stress vectors resulting in growth orientation. Not surprisingly, mutants of Gal31 are lethal [86], possibly by disrupting the 1-6-linked galactose sidechain of the AGP-Ca^2+^ glycomodule [87].

## 4. Roots 

The morphogenesis of root xylem tissues begins with the specific expression of the JIM13 AGP epitope in a single metaxylem initial just above four central cells of the quiescent centre [88]. The how and why have been dominated by the speculative idea of AGPs as signalling molecules per se. Thus, a role for AGPs in Ca^2+^-signalling was unexpected yet surprisingly close to reality, with AGP-Ca^2+^ belatedly seen here as the major dynamic source of Ca^2+^ regulated by auxin. Significantly, transformed plants that overproduce auxin show excessive xylem and phloem development [89] and bushy phenotypes [90].

**Figure 5:**
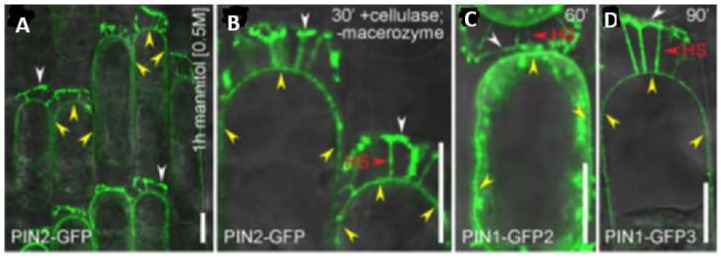
PIN proteins maintain their polarity at the cell wall of plasmolysed cells. Plasmolysis shows that apical (PIN2-GFP and PIN1-GFP-3) and basal (PIN1-GFP-2) proteins remain attached to the cell wall by their polar domains. (**A–D**) Note—yellow arrowheads = nonpolar PIN-GFP signal at the plasma membrane. HS = Hechtian strands with PIN-GFP-connections to the wall. White arrowheads = a strong persistent PIN-GFP signal at the cell wall–plasma membrane interface. Scale bars = 10 µm. Reprinted from Figure 4 of [91].

Mechanical stress patterns involved in root morphogenesis have yet to be modelled. Nevertheless, a recent comprehensive experimental approach [91] correlated gravitropism with the epidermal location of auxin efflux protein PIN2 which was dramatically co-localised with Hechtian adhesion sites between the plasma membrane and the cell wall (Figure 5).

The presence of the cell wall is essential for gravity perception [91]. These observations connect gravitropism with a rapid increase of cytosolic Ca^2+^ [51] and the following conclusion [91]: “Thus, the identified tight link between the cell wall and cell polarity provides the conceptual possibility for regulation of signal fluxes and, ultimately, plant development via signaling from the extracellular matrix.” Such evidence suggests that auxin-based mechanoperception and subsequent root morphogenesis involve the Hechtian growth oscillator. This fundamental phenomenon supersedes textbook dogma and hypotheses in highly contentious and confusing fields [49,92,93,94] that struggle to disentangle cause from effect. As a prime example, consider the challenge to identify the gravity sensor. Although quite unknown, it may involve critical components of the Hechtian oscillator as “the very earliest stages of gravitropic bending depend on auxin-stimulated Ca^2+^ influx” [41,51]*.* Nevertheless, the Cholodny–Went explanation of auxin redistribution remains unquestioned dogma [95]: “The ability of roots to reorient their growth in response to changes in the gravity vector is dependent on the asymmetric redistribution of auxin at the root tip” [41]. This demands a biochemical explanation.

The root–shoot paradox: root tip gravitropism increases auxin at the lower side that decreases growth while less auxin at the upper side increases growth. However, in shoots, a similar auxin distribution in response to gravity reverses the direction of growth. We resolve this paradox by asking: How does auxin inhibit growth at the lower side of the root? Extensin peroxidase [96] provides the answer: crosslinked extensin decreases the rate of cell extension ([97] cf. [47]) via di-isodityrosine formation [98,99]. Presumably, programmed cells of the root tip respond to high levels of auxin simply by enhancing the enzymic crosslinking of wall proteins. Thus, extensins and AGPs, as the Yin and Yang of cell extension, may illustrate yet again the ingenious parsimony of nature that involves these hydroxyproline-rich glycoproteins as both negative and positive regulators of cell extension.

**Figure 6:**
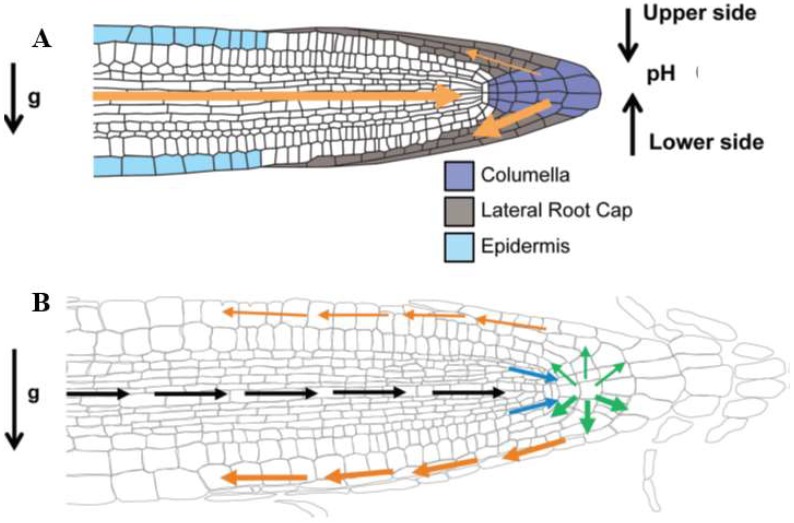
Reprinted with permission from Sato et al. [49]. Root gravitropism in *Arabidopsis*. (**A**) Gravity perception in *Arabidopsis thaliana.* At time point 0, roots grow vertically. After a 90° turn, the following events occur: (1) At 10 s, statoliths are still at the old bottom of the cell. After 3 min, statoliths move towards the new bottom of the cell to be uniformly distributed at 5 min [92]; (2) PIN3 and PIN7 relocalization is achieved 2 min after the gravity stimulus and, in consequence, a lateral auxin gradient is generated between the upper and lower side of the root (thin and thick orange arrows respectively) [100]; (3) the development of differential extracellular pH levels between the upper (acidic) and lower (alkaline) side of the gravistimulated root [51]; (**B**) Gravity signal transduction and transmission via auxin transport and redistribution. Black arrows show that AUX1 and PIN2 channel auxin from the shoot to the root tip. Blue arrows show how PIN4 distributes auxin efflux through the vascular tissue to the columella cells. PIN3 and PIN7 set up the auxin flow (green arrows), with an accumulation on the lower side of the root. PIN2 and AUX1 transport auxin through the lateral root cap to the epidermal cells in the elongation zone (orange arrows) where the actual growth response will occur.

While the above approach to a complex problem is consistent with Occam’s razor, it contrasts with the complexity of recent papers that deal with the dynamic rearrangement of PIN proteins transporting auxin from both root tip and the shoot via xylem and root epidermis (Figure 6).

However, no convincing molecular mechanism has been offered for auxin-increased cell extension. Auxin has also not been successfully connected with Ca^2+^ signalling although [49] “auxin somehow activates cytosolic Ca^2+^ waves,” the major source being the Hechtian oscillator via auxin-activated H^+^-ATPase dissociation of AGP-Ca^2+^ that releases Ca^2+^ to the cytosol via Ca^2+^ channels. How that translates into increased cell extension in the root tip elongation zone remains a conundrum, as discussed above. Although the prevalent statolith hypothesis is now textbook dogma, the precise molecular details of statolith mechanotransduction remain quite unknown. Mutants that lack amyloplast statoliths do not resolve the problem: while they decrease the gravitational response, they do not eliminate it [93]. Finally, both plasma membrane H^+^-ATPases [25] (~11 in *Arabidopsis*) and PIN proteins recycle in response to mechanotransduction, thus adding an additional level of complexity.

This raises the pertinent question: What is the biochemical source of signalling Ca^2+^ and its connection to auxin? Regions of high auxin biosynthesis, e.g., meristems, young primordia and reproductive organs, express YUCCA, a flavin mono-oxygenase that decarboxylates indole pyruvate to yield auxin [48]. Arguably, Hechtian adhesion and auxin-activated plasma membrane ATPase dissociate AGP-Ca^2+^ to provide Ca^2+^ that activates exocytosis enabling the gravity vector (9.8 N·kg^-1^) to redistribute auxin by recycling the auxin efflux PIN proteins. As the root tip derives its auxin from transport through central root tissues and also local biosynthesis (Figure 6), the columella is quite possibly the gravity sensor as it also contains the statoliths. However, that view is not consistent with the Hechtian oscillator and the Hechtian transduction of the gravity vector leading to auxin transport and its asymmetric redistribution by PIN1 and PIN3 auxin efflux proteins [26] cf. [41].

The Hechtian oscillator is also evident in mature root tissues such as the pericycle which expresses AGPs [101] and generates adventitious lateral roots and a specific extensin ([102] cf. [75]). Possibly, pericycle progenitor cell AGPs scavenge local Ca^2+^ that enhances exocytosis when stimulated by auxin supplied from root tip biosynthesis [103]. Similarly, root hair positioning is also correlated with AGP-reactive epidermal cells Figure 4A in [101], while defects in root tip auxin biosynthesis result in a shift of root hair emergence towards the shoot.

## 5. Shoots

**Figure 7:**
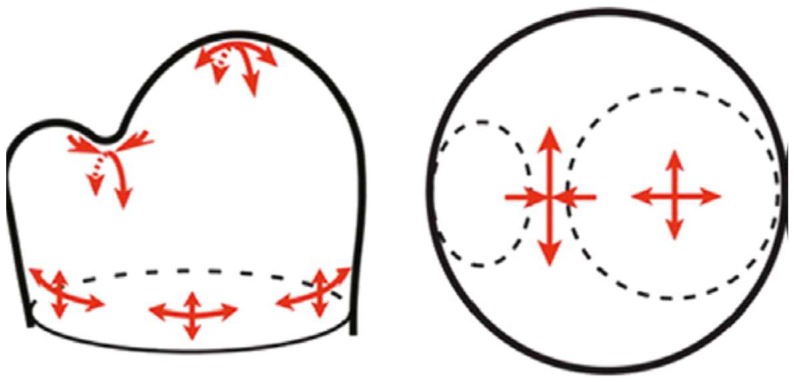
Stress vectors in the shoot apical meristem. The suggested changing meristem stress patterns cause auxin depletion in the boundary region as primordia form [26]. Note that the magnitude and direction of the stress vector are strongly anisotropic (red arrows) in the boundary region between the primordium and shoot apical meristem apex. Reprinted from [85] with permission from AAAS).

Morphogenesis of the shoot involves phyllotaxis of leaves and flowers; a problem of formidable complexity recognised by Egyptian, Greek and Roman scholars since antiquity [104] up to the present. Numerous competing hypotheses largely based on mathematical formulations connect physical forces to growth, but with many biochemical unknowns. Recent significant experimental advances [26,68,85] closely connect the stress vectors of growing tissues with the reorientation of auxin efflux PIN proteins (Figure 7): exocytosis and endocytosis (transcytosis) rapidly redeploy PIN proteins to expanding walls that show the greatest stress or strain. This implies that stress-induced signals from the cell wall promote the accumulation of PIN1 (and thus export of auxin) at the nearest membrane of cells stressed by their rapid expansion as follows.

Rapid PIN1 redeployment [105] raises the question of how cells perceive the mechanical signals that largely determine PIN1 polarity. In plants, the gap between biophysical stress and biochemical response is generally ignored. However, PIN protein recycling depends on Ca^2+^-directed vectorial exocytosis, and therefore shares essential components of the Hechtian growth oscillator that translate biophysical force into biochemistry. This connects the oscillator with the generation of new primordia; hypothetically, their regular spatial separation or phyllotaxis results from competing demands for auxin and Ca^2+^ morphogens, channelled to the most rapidly growing cells by PIN proteins and cell surface AGPs that scavenge Ca^2+^. This results in auxin depletion and slower growth in the boundary or “saddle region” between the apical meristem and primordia. Indeed, competition for auxin and Ca^2+^ may determine the angle of divergence between successive primordia and thus the creation of whorls [106], although the morphogenesis of flowering involving, for example, a protein florigen [107] and development of seeds is beyond the scope of this brief essay.

Auxin initiates phyllotaxis by transport against a concentration gradient to sites of primordia formation; these depend on the polarity of auxin efflux (PIN) proteins maintained by the cell wall [91,108]. However, the apical dominance of the shoot, although generally ascribed to the auxin inhibition of lateral bud growth, may actually depend on the absence of PIN polarity from dormant tissues. Arguably, the removal of the shoot apex restores PIN1 polarisation in the bud [109] via renewed axillary auxin transport that breaks dormancy. A role for the Hechtian growth oscillator in apical dominance seems likely and is consistent with the bushy phenotype of tomato plants over-expressing AGP LeAGP1 [110]. The size of the AGP-Ca^2+^ capacitor could determine the balance between the dormancy and active growth of axillary buds.

## 6. The Evolution of Morphogenesis

Phylogeny and morphogenesis are inextricably linked [111], and so our brief description can only attempt a broad-brush summary that reconciles old observations with new discoveries. Specifically, these relate wall stress–strain and Hechtian adhesion to the control of cytosolic Ca^2+^ by auxin. Thus, auxin efflux PIN proteins transport auxin to target sites, where the activation of H^+^-ATPase releases Ca^2+^ from AGP-Ca^2+^, triggering exocytosis. This paradigm shift can be traced back to the primordial soup and unifies the chemiosmotic proton motive force with auxin and Ca^2+^ signalling. By analogy with inscriptions on the Rosetta stone, once decoded, these three fundamental transport phenomena translate the same growth message into morphogenesis.

Morphogenesis begins with prebiotic synthesis from CH_4_, CO_2_, and water to yield HCHO [112], a precursor to simple sugars via the Butlerov (formose) reaction. The subsequent prebiotic generation of self-replicating molecules and self-assembling amphiphiles formed lipid membranes and nanobubble aerosols. Such semipermeable membrane-bounded protocells sequestered and concentrated reactants by chemiosmosis, most likely in Darwin’s “warm little pond” (In a letter to J.D. Hooker 1871). Hence, the origin of Peter Mitchell’s proton motive force [70], a universal phenomenon that precedes RNA-DNA replication and drives reactions that exploit the chemistry in a watery environment of planets in the “Goldilocks zone”. The first simple prokaryotes were chemical machines; their further specialisation and morphological adaption led to unicellular eukaryotes, morphogenetic machines characterised by increasing structural adaptations and complexity, exemplified by the invention of chromosomes as Darlington’s “little packets of predestination”, internal membrane systems such as ER (endoplasmic reticulum), Golgi, and cell walls to protect the plasma membrane. Indeed, most major evolutionary advances from chemiosmotic energy transduction to cellular organelles appeared very early and remain as essential features of modern eukaryotic cells whose precise origins by endosymbiont capture [113] remain to be elucidated.

**Figure 8:**
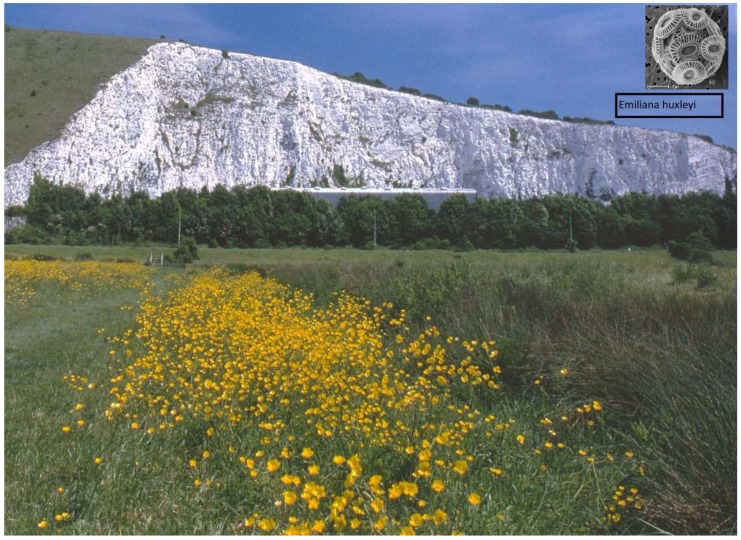
Chilly Brook chalk cliffs with *E. huxleyi*. Coccolithophores such as *E. huxleyi* (inset) constructed massive soft chalk deposits of calcium carbonate during the cretaceous period (145–65 MYA), now seen here from across the Chilly Brook water meadows dominated by *Ranunculus acris*, adjacent to the River Ouse in the South Downs National Park at Lewes, UK. This view encapsulates the entire evolutionary progression from the simplest unicellular protists such as *E. huxleyi* to advanced dicots such as *R. acris* at the pinnacle of alternation of generations, all dependent on calcium. (Photo: DTAL).

From the cell wall viewpoint, the unicellular flagellate *Chlamydomonas* provides the most striking example of evolutionary conservation and diversification, with a large complement of 182 different proteins related to hydroxyproline-rich extensins [114]. The *Chlamydomonas* wall is devoid of cellulose and consists almost entirely of glycoproteins that self-assemble as a crystal lattice [115], a remarkable precursor to their conserved self-assembly role in higher plants [84]. The leap to multicellular cooperation involved two distinct evolutionary lines: one line embedded cells in a hydroxyproline-rich glycoprotein (HRGP) protective matrix typified by volvocine algae; the other Chlorophycean line evolved silicified or calcified protective cell walls (Figure 8) typified by the calcareous plates of Coccolithophores, such as *Emiliana huxleyi* of the cretaceous that built the chalk hills and cliffs of the present era.

Static cell surface CaCO_3_ coccoliths of Coccolithophores represented by *E. huxleyi* are the probable evolutionary precursor to dynamic cell surface AGP-Ca^2+^, hence Ca^2+^ signalling. Algae increased the tensile strength of a primary cell wall prototype by co-opting cellulose, thus enabling turgor pressure and the major evolutionary pathway to metaphytes. Indeed, the composition of algal pectin-rich cellulosic walls is remarkably similar to those defined as primary walls in the cambial tissues of higher plants [116]. That raises the question: When did the Hechtian oscillator emerge? Based on the presence of Hechtian strands associated with rapid tip growth [6], we extrapolated Mitchell’s original chemiosmotic hypothesis to include Ca^2+^ signalling. This implies incredible evolutionary conservation and an early origin of the Hechtian pscillator in unicellular Chlorophycean algae such as Closterium [22] and Penium [23], as both exhibit tip growth with prominent Hechtian strands (Figure 1). 

**Figure 9:**
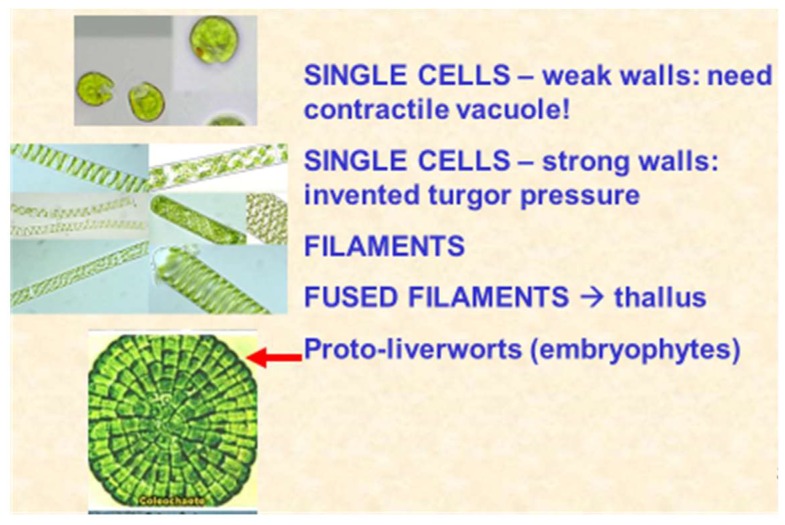
Metaphyte origins. Progression from single cells to filaments that align, forming a flat thallus. The examples here are the biflagellate *Chlamydomonas*, *Spirogyra* and the red arrow points to *Coleochaete scutata*.

Single cells remaining attached after cytokinesis evolved into simple linear filamentous algae as the first step towards multicellularity and its regulation. Although auxin is a key growth regulator in higher plants, it is evident as a biosynthetic pathway in the simplest unicellular chlorophytes [117]. However, auxin transport also involves efflux PIN proteins, and these first appear in filamentous algae—“living fossils” such as *Spirogyra* [117]—with anchoring rhizoids that differentiate only from terminal cells [118]. Thus, the PIN proteins and polarity of *Spirogyra* rhizoids, as well as stretch-activated Ca^2+^ channels, nicely represent the earliest evolutionary progression from marine via freshwater to land plants (Figure 9).

**Figure 10:**
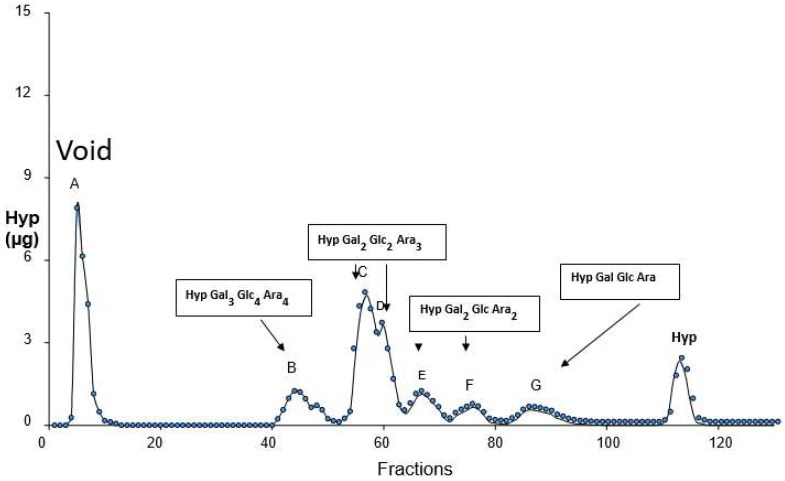
*Coleochaete* hydroxyproline glycosides. Alkaline hydrolysates of *Coleochaete scutata* cell walls fractionated on a cation exchange column gave a Hyp-glycoside profile that appeared similar to the profile of cell walls isolated from higher plants. However, closer inspection and quantitative sugar analyses showed a striking difference; in higher plants, the Hyp-glycosides are simple arabinosides, whereas *Coleochaete* shows heterooligosaccharides of up to seven sugar residues that include galactose, glucose and arabinose, compositions remarkably similar to the profile of cell walls isolated from *Chlamydomonas* [119] but different from other members of the plant kingdom [120]. Presented at the 11th Cell Wall Meeting, Copenhagen [121].

Further lateral cohesion between adjacent filaments forms a prototype thallus. Other “living fossils” include *Coleochaete* a prototype leafy liverwort (Hepaticae) that retains the fertilised diploid egg cell until “germination”. *Coleochaete* cell walls are also significantly enriched in hydroxyproline-rich glycoproteins. Alkaline hydrolysis yields a hydroxyproline glycoside profile (Figure 10) dominated by small hydroxyproline heterooligosaccharides, similar to the profile of *Chlamydomonas*, but surprisingly different from the simple Hyp-arabinoside profile of higher plants.

Although more popular notions suggest Chara or Nitella as a land plant precursor [122], that seems unlikely for several reasons; firstly, despite being invoked as a model system, their cell walls lack hydroxyproline-rich cell wall proteins; secondly, the relatively thick walls of Chara are rich in non-methyl esterified pectin; and finally, the large cells are coenocytes but remain locked in an evolutionary cul-de-sac.

**Figure 11:**
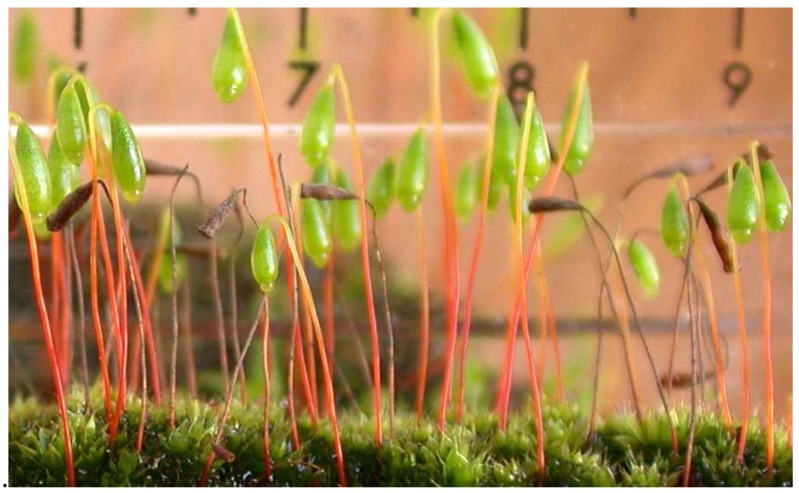
The moss *Bryum capillare* exemplifies the alternation of generations. *Bryum capillare* with attached diploid sporophytes dependent on the haploid gametophyte generation. (Photo: DTAL).

Bryophytes initiate the gametophyte–sporophyte “alternation of generations” brilliantly elucidated by Hofmeister [123] (Figure 11). 

Further evolutionary development of the attached sporophyte (e.g., with stomata) culminated in its complete dominance in higher plants with their increasingly complex division into root, shoots and leaves. Root-like structures appeared as rhizoids in the Bryophytes, but true roots defined by their gravitropic response, endogenous branching, root hairs, and a protective root cap originate from the suspensor hypophysis during angiosperm embryogenesis. While sharing much in common with true shoots, roots clearly lack many shoot characteristics and have therefore been described as “partial shoots” [72] that evolved as an adaptation to a subterranean life [124].

The ultimate reduction of the gametophyte generation to just a few cells, namely pollen and the ovule as an integumented megasporangium, completes this brief summary based on a novel growth oscillator unifying other growth regulators that control Ca^2+^ release and exocytosis. 

## 7. Postscript

Ion transport dominates this paper, which is greatly influenced (1955–1961) by personal acquaintance (DTAL) with the Cambridge pioneers who included Peter Mitchell (proton pump), David Keilin (cytochrome discovery) and Robert “Robin” Hill Reaction (photolysis of water). The cell wall protein was undoubtedly inspired by Fred Sanger in the adjacent lab (protein structure, 1958 Nobel Prize), and Joseph E. Varner [125] on his sabbatical leave acted as a “midwife to wall protein.” H.A. Krebs was one of DTAL’s BA examiners. Two years of National Service as a corporal radio instructor at the Royal Air Force Radio School Yatesbury (1953–1955) inspired thoughts about biological oscillators. Finally, the poet Alexander Pope (1688–1744) summarises the effect of the intellectual environment exemplified by Joseph Needham (morphogenesis) and Don Northcote (cell walls) in the Cambridge Department of Biochemistry: “As the twig is bent so is the tree inclined.”

## Figures and Tables

**Figure 3:**
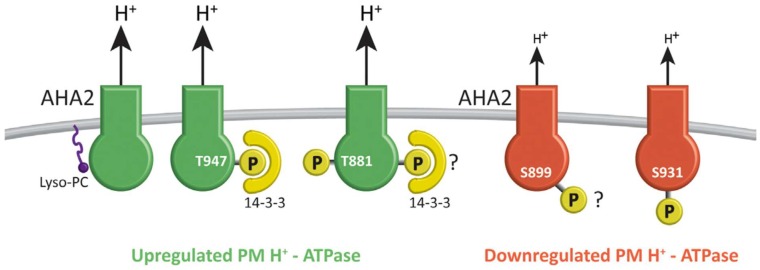
Posttranslational regulation of the PM H^+^-ATPase alternates between two states: reprinted from [38]. Red = downregulated pumps hydrolyze ATP with low efficiency, hence the low transport of H^+^. Green = upregulated pumps with high ATPase efficiency, and high H^+^ transport rates. C-terminal regulatory domains control transition between the two states. The phosphorylation (P) of the C-terminal penultimate threonine residue (Thr-947 in pump AHA2) creates a binding site for a 14-3-3 protein that stabilises the pump. The binding of lysophosphatidylcholine (Lyso-PC) and phosphorylation at Thr-881 also activates the PM H^+^-ATPase independently of phosphorylation and 14-3-3 protein binding. It is not known whether phosphorylation at Thr-881 in the C-terminal domain interferes with or depends on 14-3-3 binding (as indicated by the question mark). Phosphorylation at Ser-899 or Ser-931 inactivates the pump. Phosphorylation at Ser-931 blocks the binding of 14-3-3 protein, but is not known for Ser-899. Only well-characterized regulatory events are shown.

**Table 1:** Piconewton forces crucial to biochemical function and Hechtian adhesion.

System	Action	Adhesion Strength pN	References
Integrin-RGD binding	Binding	~10	[35]
GPI-proteins	Adhesion	103–350 pN	[31]
Protein e.g., talin	Unfolding	5 pN	[32]
Cation channels	Open	4 pN (to open)	[36]

**Table 2:** The PIN Ffamily of auxin efflux proteins.

Auxin Efflux Protein	Major Location or Function	Efflux Orientation	References
PIN 1	Outer protoderm and lower cells of proembryo	Rootward	[71]
PIN 2	Root epidermis and lateral root cap; regulates gravitropism	Shootward	[26] [42]
PIN 3	Gravitropism	Rootward	[26]
PIN 4	Hypophysis		[71]
PIN 5	ER	Cytosol	[42]
PIN 6	ER	Cytosol	[42]
PIN 7	Suspensor	Apical towards embryo Rootward	[80]
PIN 8	ER	cytosol	[42]

## References

[1] Lamport D.T.A. (1963). The Primary Cell Wall.

[2] Turing A.M. (1952). The chemical basis of morphogenesis. Philos. Trans. R. Soc. Lond. B.

[3] Thompson D.W. (1917). On Growth and Form.

[4] Wardlaw C.W. (1952). Phylogeny and Morphogenesis.

[5] Needham J. (1942). Biochemistry and Morphogenesis.

[6] Lamport D.T.A., Tan L., Held M.A., Kieliszewksi M.J. (2018). Pollen tube growth and guidance: Occam's razor sharpened on a molecular arabinogalactan glycoprotein Rosetta Stone. New Phytol..

[7] Tan L., Qiu F., Lamport D.T.A., Kieliszewski M.J. (2004). Structure of a hydroxyproline-arabinogalactan polysaccharide from repetitive Ala-Hyp expressed in transgenic *Nicotiana tabacum*. J. Biol. Chem..

[8] Tan L., Varnai P., Lamport D.T.A., Yuan C., Xu J., Qiu F., Kieliszewski M.J. (2010). Plant O-Hydroxyproline Arabinogalactans Are Composed of Repeating Trigalactosyl Subunits with Short Bifurcated Side Chains. J. Biol. Chem..

[9] Aspinall G.O., Malloy J.A., Craig J.W.T. (1969). Extracellular polysaccharides from suspension-cultured sycamore cells. Can. J. Biochem..

[10] Lamport D.T.A. (1970). Cell wall metabolism. Ann. Rev. Plant Physiol..

[11] Lamport D.T.A., Northcote D.H. (1960). Hydroxyproline in primary cell walls of higher plants. Nature.

[12] Lamport D.T.A., Varnai P. (2013). Periplasmic arabinogalactan glycoproteins act as a calcium capacitor that regulates plant growth and development. New Phytol..

[13] Johnson K.L., Cassin A.M., Lonsdale A., Bacic A., Doblin M., Schultz C.J. (2017). A Motif and Amino Acid Bias Bioinformatics Pipeline to Identify Hydroxyproline-Rich Glycoproteins. Plant Physiol..

[14] Ma Y., Yan C., Li H., Wu W., Liu Y., Wang Y., Chen Q., Ma H. (2017). Bioinformatics Prediction and Evolution Analysis of Arabinogalactan Proteins in the Plant Kingdom. Front. Plant Sci..

[15] Pennell R.I., Roberts K. (1990). Sexual development in the pea is presaged by altered expression of arabinogalactan protein. Nature.

[16] Pennell R.I., Janniche L., Scofield G.N., Booij H., de Vries S.C., Roberts K. (1992). Identification of a Transitional Cell State in the Developmental Pathway to Carrot Somatic Embryogenesis. J. Cell Biol..

[17] Showalter A.M. (2001). Arabinogalactan-proteins: Structure, expression and function. Cell. Mol. Life Sci..

[18] Coimbra S., Jones B., Pereira L.G. (2008). Arabinogalactan proteins (AGPs) related to pollen tube guidance into the embryo sac in Arabidopsis. Plant Signal. Behav..

[19] Hepler P.K. (2016). The Cytoskeleton and Its Regulation by Calcium and Protons. Plant Physiol..

[20] Hecht K. (1912). Studien uber den Vorgang der Plasmolyse.

[21] Pont-Lezica R.F., McNally J.G., Pickard B.G. (1993). Wall-to-membrane linkers in onion epidermis: Some hypotheses. Plant Cell Environ..

[22] Domozych D.S., Roberts R., Danyow C., Flitter R., Smith B. (2003). Plasmolysis, Hechtian strand formation, and localized membrane wall adhesions in the desmid *Closterium acerosum* (Chlorophyta). J. Phycol..

[23] Raimundo S.C., Sorensen I., Tinaz B., Ritter B., Rose J.K.C., Domozych D.S. (2018). Isolation and manipulation of protoplasts from the unicellular green alga *Penium margaritaceum*. Plant Methods.

[24] Buer C.S., Weathers P.J., Swartzlander G.A. (2000). Changes in Hechtian strands in cold-hardened cells measured by optical microsurgery. Plant Physiol..

[25] Hashimoto-Sugimoto M., Higaki T., Yaeno T., Nagami A., Irie M., Fujimi M., Miyamoto M., Akita K., Negi J., Shirasu K. (2013). A Munc13-like protein in Arabidopsis mediates H^+^-ATPase translocation that is essential for stomatal responses. Nat. Commun..

[26] Sampathkumar A., Yan A., Krupinski P., Meyerowitz E.M. (2014). Physical Forces Regulate Plant Development and Morphogenesis. Curr. Biol..

[27] Serpe M.D., Nothnagel E.A. (1999). Arabinogalactan-proteins in the multiple domains of the plant cell surface. Adv. Bot. Res..

[28] Oxley D., Bacic A. (1999). Structure of the glycosylphosphatidylinositol anchor of an arabinogalactan protein from Pyrus communis suspension-cultured cells. Proc. Natl. Acad. Sci. USA.

[29] Eisenhaber B., Wildpaner M., Schultz C.J., Borner G.H.H., Dupree P., Eisenhaber F. (2003). Glycosylphosphatidylinositol lipid anchoring of plant proteins. Sensitive prediction from sequence- and genome-wide studies for Arabidopsis and rice1. Plant Physiol..

[30] Borner G.H.H., Lilley K.S., Stevens T.J., Dupree P. (2003). Identification of glycosylphosphatidylinositol-anchored proteins in Arabidopsis. A proteomic and genomic analysis. Plant Physiol..

[31] Cross B., Ronzon F., Roux B., Rieu J.P. (2005). Measurement of the Anchorage Force between GPI-Anchored Alkaline Phosphatase and Supported Membranes by AFM Force Spectroscopy. Langmuir.

[32] Dufort C.C., Paszek M.J., Weaver V.M. (2011). Balancing forces: Architectural control of mechanotransduction. Nat. Rev. Mol. Cell Biol..

[33] Murthy S.E., Dubin A.E., Patapoutian A. (2017). Piezos thrive under pressure: Mechanically activated ion channels in health and disease. Nat. Rev. Mol. Cell Biol..

[34] Tan L., Eberhard S., Pattathil S., Warder C., Glushka J., Yuan C., Hao Z., Zhu X., Avci U., Miller J.S. (2013). An Arabidopsis Cell Wall Proteoglycan Consists of Pectin and Arabinoxylan Covalently Linked to an Arabinogalactan Protein. Plant Cell.

[35] Moore S.W., Roca-Cusachs P., Sheetz M.P. (2010). Stretchy Proteins on Stretchy Substrates: The Important Elements of Integrin-Mediated Rigidity Sensing. Dev. Cell.

[36] Sukharev S., Sachs F. (2012). Molecular force transduction by ion channels—Diversity and unifying principles. J. Cell Sci..

[37] Yusko E.C., Asbury C.L. (2014). Force is a signal that cells cannot ignore. Mol. Biol. Cell.

[38] Falhof J., Pedersen J.T., Fuglsang J.T., Palmgren M. (2016). Plasma Membrane H^+^-ATPase Regulation in the Center of Plant Physiology. Mol. Plant.

[39] Pacheco-Villalobos D., Diaz-Moeeno M., van der Schuren A., Tamaki T., Kang Y.H., Gujas B., Novak O., Jaspert N., Li Z., Wolf S. (2016). The Effects of High Steady State Auxin Levels on Root Cell Elongation in Brachypodium. Plant Cell.

[40] Cieslak M., Runions A., Prusinkiewicz P. (2015). Auxin-driven patterning with unidirectional fluxes. J. Exp. Bot..

[41] Leyser O. (2018). Auxin Signaling. Plant Physiol..

[42] Habets M.E., Offringa R. (2014). PIN-driven polar auxin transport in plant developmental plasticity: A key target for environmental and endogenous signals. New Phytol..

[43] Hayashi Y., Takahashi K., Inoue S., Kinoshita T. (2014). Abscisic Acid Suppresses Hypocotyl Elongation by Dephosphorylating Plasma Membrane H^+^-ATPase in *Arabidopsis thaliana*. Plant Cell Physiol..

[44] De Vries J., Curtis B.A., Gould S.B., Archibald J.M. (2018). Embryophyte stress signaling evolved in the algal progenitors of land plants. Proc. Natl. Acad. Sci. USA.

[45] Folta K.M., Lieg E.G., Durham T., Spalding E.P. (2003). Primary Inhibition of Hypocotyl Growth and Phototropism Depend Differently on Phototropin Mediated Increases in Cytoplasmic Calcium Induced by Blue Light. Plant Physiol..

[46] Zhao Y., Qi Z., Berkowitz G.A. (2013). Teaching an Old Hormone New Tricks: Cytosolic Ca^2+^ Elevation Involvement in Plant Brassinosteroid Signal Transduction Cascades. Plant Physiol..

[47] Swarup R., Perry P., Hagenbeek D., Van Der Straeten D., Beemster G.T.S., Sandberg G., Bhalerao R., Ljung K., Bennett M. (2007). Ethylene Upregulates Auxin Biosynthesis in Arabidopsis Seedlings to Enhance Inhibition of Root Cell Elongation. Plant Cell.

[48] Muday G.K., Rahman A., Binder B.M. (2012). Auxin and ethylene: Collaborators or competitors?. Trends Plant Sci..

[49] Sato E.M., Hijazi H., Bennett M.J., Vissenjberg K., Swarup R. (2015). New insights into root gravitropic signaling. J. Exp. Bot..

[50] Okada K., Ito T., Fukazawa J., Takahashi Y. (2017). Gibberellin Induces an Increase in Cytosolic Ca^2+^ via a DELLA-Independent Signaling Pathway. Plant Physiol..

[51] Monshausen G.B., Miller N.D., Murphy A.S., Gilroy J.S. (2011). Dynamics of auxin-dependent Ca^2+^ and pH signaling in root growth revealed by integrating high-resolution imaging with automated computer vision-based analysis. Plant J..

[52] Yamazaki C., Fujii N., Miyazawa Y., Kamada M., Kasahara H., Osada I., Shimazu T., Fusejima Y., Higashibata A., Yamazaki T. (2016). The gravity-induced re-localization of auxin efflux carrier CsPIN1 in cucumber seedlings: Spaceflight experiments for immunohistochemical microscopy. Microgravity.

[53] Domingos P., Prado A.M., Wong A., Gehring C., Feijo J.A. (2015). Nitric Oxide: A Multitasked Signaling Gas in Plants. Mol. Plant.

[54] Pucciariello O., Legris M., Rojas C.C., Iglesias M.J., Hernando C.E., Dezar C., Vazquez M., Yanovsky M.J., Finlayson S.A., Prat S. (2018). Rewiring of auxin signaling under persistent shade. Proc. Natl. Acad. Sci. USA.

[55] Foreman J., Demidchik V., Bothwell J.H.F., Mylona P., Miedema H., Torres M.A., Linstead P., Costa S., Brownlee C., Jones J.D.G. (2003). Reactive oxygen species produced by NADPH oxidase regulate plant cell growth. Nature.

[56] Proseus T.E., Boyer J.S. (2006). Calcium pectate chemistry controls growth rate of *Chara coralline*. J. Exp. Bot..

[57] Levesque-Tremblay G., Pelloux J., Braybrook S.A., Muller K. (2015). Tuning of pectin methylesterification: Consequences for cell wall biomechanics and development. Planta.

[58] Van den Bulck K., Swennen K., Loosveld A.M., Christophe M., Brijs K., Proost P., Van Damme J., Campenhout S., Mort A., Delcour J.A. (2005). Isolation of cereal arabinogalactan-peptides and structural comparison of their carbohydrate and peptide moieties. J. Cereal Sci..

[59] Braybrook S.A., Peaucell A. (2013). Mechano-Chemical Aspects of Organ Formation in Arabidopsis thaliana: The Relationship between Auxin and Pectin. PLoS ONE.

[60] Cosgrove D.J. (2016). Catalysts of plant cell wall loosening. F100Research.

[61] Cosgrove D.J. (2018). Diffuse Growth of Plant Cell Walls. Plant Physiol..

[62] Heyn A.N.J. (1940). The physiology of cell elongation. Bot. Rev..

[63] Ding J.P., Pickard B.G. (1993). Mechanosensory calcium-selective cation channels in epidermal cells. Plant J..

[64] Takahashi K., Hayashi K., Kinoshita T. (2012). Auxin Activates the Plasma Membrane H^+^-ATPase by Phosphorylation during Hypocotyl Elongation in Arabidopsis. Plant Physiol..

[65] Mazhab-Jafari M.T., Rohou A., Schmidt C., Bueler S.A., Benlekbir S., Robinson C.V., Rubinstein J.L. (2016). Atomic model for the membrane-embedded VO motor of a eukaryotic V-ATPase. Nature.

[66] Lane N. (2005). Power, Sex, Suicide: Mitochondria and the Meaning of Life.

[67] Lamport D.T.A., Kieliszewksi M.J., Showalter A.M. (2006). Salt-stress upregulates periplasmic arabinogalactan-proteins: Using salt-stress to analyse AGP function. New Phytol..

[68] Heisler G., Ohno C., Das P., Sieber P., Reddy G.V., Long J.A., Meyerowitz E.M. (2005). Patterns of Auxin Transport and Gene Expression during Primordium Development Revealed by Live Imaging of the *Arabidopsis* Inflorescence Meristem. Curr. Biol..

[69] Rayle D.L., Cleland R.E. (1992). The acid growth theory of auxin-induced cell elongation is alive and well. Plant Physiol..

[70] Mitchell P. (1961). Coupling of phosphorylation to electron and hydrogen transfer by a chemi-osmotic type of mechanism. Nature.

[71] Moller B., Weijers D. (2009). Auxin Control of Embryo Patterning. Cold Spring Harb. Perspect. Biol..

[72] Arber A. (1950). The Natural Philosophy of Plant Form.

[73] Steward F.C., Mapes M.O., Smith J. (1958). Growth and organised development of cultured cells. I. Growth and division of freely suspended cells. Am. J. Bot..

[74] Steward F.C., Mapes M.O., Mears K. (1958). Growth and organised development of cultured cells. II Organisation in cultures grown from freely suspended cells. Am. J. Bot..

[75] Dolan L., Janmaat K., Willemsen V., Linstead P., Poethig S., Roberts K., Scheres B. (1993). Cellular organisation of the *Arabidopsis thaliana* root. Development.

[76] Kidner C., Sundaresan V., Roberts K., Dolan L. (2000). Clonal analysis of the Arabidopsis root confirms that position, not lineage, determines cell fate. Planta.

[77] Qin Y., Zhao J. (2006). Localization of arabinogalactan proteins in egg cells, zygotes, and two-celled proembryos and effects of β-D-glucosyl Yariv reagent on egg cell fertilization and zygote division in *Nicotiana tabacum* L.. J. Exp. Bot..

[78] Rubery P.H., Sheldrake A.R. (1974). Carrier-mediated Auxin Transport. Planta.

[79] Okada K., Ueda J., Komaki M.K., Bell C.J., Shimura Y. (1991). Requirement of the auxin polar transport system in early stages of Arabidopsis floral bud formation. Plant Cell.

[80] Peng X., Sun M.X. (2018). The suspensor as a model system to study the mechanism of cell fate specification during early embryogenesis. Plant Reprod..

[81] Pennell R.I., Janniche L., Kjellbom P., Scofield G.N., Peart J.M., Roberts K. (1991). Developmental regulation of a plasma membrane arabinogalactan protein epitope in oilseed rape flowers. Plant Cell.

[82] Pickett-Heaps J.D., Northcote D.H. (1966). Organisation of microtubules and endoplasmic reticulum during mitosis and cytokinesis in wheat meristems. J. Cell Sci..

[83] Schaefer E., Belcram K., Uyttewaal M., Duroc Y., Goussot M., Legland D., Laruelle O., de Tauzia-Moreau M.L., Pastuglia M., Bouchez D. (2017). The preprophase band of microtubules controls the robustness of division orientation in plants. Science.

[84] Cannon M.C., Terneus K., Hall Q., Wang Y., Wegenhart B.L., Chen L., Lamport D.T.A., Chen Y., Kieliszewski M.J. (2008). Self-assembly of the plant cell wall requires an extensin scaffold. Proc. Natl. Acad. Sci. USA.

[85] Hamant O., Heisler M.G., Jonnson H., Krupinski P., Uyttewaal M., Bokov P., Corson F., Sahlin P., Boudaoud A., Meyerowitz E.M. (2008). Developmental patterning by mechanical signals in *Arabidopsis*. Science.

[86] Geshi N., Johansen J.N., Dilokpimol A., Rolland A., Belcram K., Verger S., Kotake T., Tsumuraya Y., Kaneko S., Tryfona T. (2013). A galactosyltransferase acting on arabinogalactan protein glycans is essential for embryo development in Arabidopsis. Plant J..

[87] Basu D., Liang Y., Liu X., Himmeldirk K., Faik A., Kieliszewski M., Held M.A., Showalter A.M. (2013). Functional identification of a hydroxyproline-*O*-galactosyltransferase specific for arabinogalactan protein biosynthesis in *Arabidopsis*. J. Biol. Chem..

[88] Dolan L., Linstead P., Roberts K. (1995). An AGP epitope distinguishes a central metaxylem initial from other vascular initials in the Arabidopsis root. Protoplasma.

[89] Olatunji D., Geelen D., Verstraeten I. (2017). Control of Endogenous Auxin Levels in Plant Root Development. Int. J. Mol. Sci..

[90] Sun W., Kieliszewski M.J., Showalter A.M. (2004). Overexpression of tomato LeAGP-1 arabinogalactan-protein promotes lateral branching and hampers reproductive development. Plant J..

[91] Feraru E., Feraru M.I., Kleine-Vehn J., Martinie A., Mouille G., Vanneste S., Vernhettes S., Runions J., Friml J. (2011). PIN Polarity Maintenance by the Cell Wall in Arabidopsis. Curr. Biol..

[92] Leitz G., Kang B.H., Schoenwaelder M.E.A., Staehelin L.A. (2009). Statolith Sedimentation Kinetics and Force Transduction to the Cortical Endoplasmic Reticulum in Gravity-Sensing Arabidopsis Columella Cells. Plant Cell.

[93] Caspar T., Pickard B.G. (1989). Gravitropism in a starchless mutant of Arabidopsis. Planta.

[94] Berut A., Chauvet H., Legue V., Moulia B., Pouliquen V., Forterre Y. (2018). Gravisensors in plant cells behave like an active granular liquid. Proc. Natl. Acad. Sci. USA.

[95] Firn R.D., Wagstaff C., Digby J. (2000). The use of mutants to probe models of gravitropism. J. Exp. Bot..

[96] Everdeen D.S., Kiefer S., Willard J.J., Muldoon E.P., Dey P.M., Li X.B., Lamport D.T.A. (1988). Enzymic crosslinkage of monomeric extensin precursors *in vitro*. Plant Physiol..

[97] Cleland R.E., Karlsnes A. (1967). A possible role for hydroxyproline-containing proteins in the cessation of cell elongation. Plant Physiol..

[98] Epstein L., Lamport D.T.A. (1984). An intramolecular linkage involving isodityrosine in extension. Phytochemistry.

[99] Held M.A., Tan L., Kamyab A., Hare M., Shpak E., Kieliszewksi M.J. (2004). Di-isodityrosine is the intermolecular cross-link of isodityrosine-rich extensin analogs cross-linked *in vitro*. J. Biol. Chem..

[100] Friml J., Wisniewski J.P., Benkova E., Mengen K., Palme K. (2002). Lateral relocation of auxin efflux regulator PIN3 mediates tropism in Arabidopsis. Nature.

[101] Knox J.P., Day S., Roberts K. (1989). A set of cell surface glycoproteins forms an early marker of cell position but not cell type, in the root apical meristem of *Daucus carota* L.. Development.

[102] Keller B., Lamb C.J. (1989). Specific expression of a novel cell wall hydroxyproline-rich glycoprotein gene in lateral root initiation. Genes Dev..

[103] Ikeda Y., Men S., Fischer U., Stepanova A.N., Alonso J.M., Ljung K., Grebe M. (2009). Local auxin biosynthesis modulates gradient-directed planar polarity in *Arabidopsis*. Nat. Cell Biol..

[104] Adler I., Barabe D., Jean R.V. (1997). A History of the Study of Phyllotaxis. Ann. Bot..

[105] Vernoux T., Besnard F., Traas J. (2010). Auxin at the Shoot Apical Meristem. Cold Spring Harb. Perspect. Biol..

[106] Coen E.S., Meyerowitz E.M. (1991). The war of the whorls: Genetic interactions controlling flower development. Nature.

[107] Taoka K., Ohki I., Tsuji H., Kojima C., Shimamoto K. (2013). Structure and function of florigen and the receptor complex. Trends Plant Sci..

[108] Luschnig C., Vert G. (2014). The dynamics of plant plasma membrane proteins: PINs and beyond. Development.

[109] Balla J., Medvedova Z., Kalousek P., Matiješèuková N., Friml J., Reinohl V., Prochazka S. (2016). Auxin flow-mediated competition between axillary buds to restore apical dominance. Sci. Rep..

[110] Zhao Z.D., Tan L., Showalter A.M., Lamport D.T.A., Kieliszewski M.J. (2002). Tomato LeAGP-1 arabinogalactan-protein purified from transgenic tobacco corroborates the Hyp contiguity hypothesis. Plant J..

[111] Wardlaw C.W. (1955). Evidence relating to the diffusion reaction theory of morphogenesis. New Phytol..

[112] Jalbout A.F., Leif A., Adamowicz L., Polt R., Apponi A.J., Ziurys L.M. (2007). Sugar Synthesis from a Gas-Phase Formose Reaction. Astrobiology.

[113] Margulis L. (1970). Origin of Eukaryotic Cells.

[114] Liu X., Wolfe R., Welch L.R., Domozych D.S., Popper Z.A., Showalter A.M. (2016). Bioinformatic Identification and Analysis of Extensins in the Plant Kingdom. PLoS ONE.

[115] Roberts K. (1974). Crystalline glycoprotein cell walls of algae: Their structure, composition and assembly. Philos. Trans. R. Soc. Lond. B.

[116] Kerr T., Bailey I.W. (1934). The cambium and its derivative tissues. X Structure, optical properties and chemical composition of the so-called middle lamella. J. Arnold Arbor..

[117] De Smet I., Voss U., Lau S., Wilson M., Shao N., Timme R.E., Swarup R., Kerr I., Hodgman C., Bock R. (2010). Unraveling the Evolution of Auxin Signaling. Plant Physiol..

[118] Inoue N., Yamada S., Nagata Y., Shimmen T. (2002). Rhizoid Differentiation in Spirogyra: Position Sensing by Terminal Cells. Plant Cell Physiol..

[119] Miller D.H., Lamport D.T.A., Miller M. (1972). Hydroxyproline heterooligosaccharides in *Chlamydomonas*. Science.

[120] Lamport D.T.A., Miller D.H. (1971). Hydroxyproline arabinosides in the plant kingdom. Plant Physiol..

[121] Buglass S., Lamport D.T.A., Xu J., Tan L., Kieliszewksi M.J. Origin of the Land Plants: Is *Coleochaete* their closest living relative? The writing is on the wall. Proceedings of the 11th Cell Wall Meeting.

[122] Delwiche C.F., Cooper E.D. (2015). The Evolutionary Origin of a Terrestrial Flora. Curr. Biol..

[123] Kaplan D.R., Cooke T.J. (1996). The genius of Wilhelm Hofmeister: The origin of causal-analytical research in plant development. Am. J. Bot..

[124] Kenrick P., Strullu-Derrien C. (2014). The Origin and Early Evolution of Roots. Plant Physiol..

[125] Chrispeels M.J., Varner J.E. (1997). Biographical Memoirs.

